# Genome-Wide Identification of *GATA* Family Genes and Functional Analysis of *IbGATA17* Under Drought Stress in Sweetpotato

**DOI:** 10.3390/genes16101237

**Published:** 2025-10-19

**Authors:** Yinghui Yang, Ruitao Liu, Qingchang Liu, Shaozhen He, Shaopei Gao, Huan Zhang, Ning Zhao, Hong Zhai

**Affiliations:** Key Laboratory of Sweetpotato Biology and Biotechnology, Ministry of Agriculture and Rural Affairs/Beijing Key Laboratory of Crop Genetic Improvement/Laboratory of Crop Heterosis and Utilization, Ministry of Education, College of Agronomy and Biotechnology, China Agricultural University, Beijing 100193, China

**Keywords:** sweetpotato, *GATA* gene family, evolutionary analysis, expression pattern analysis, function analysis

## Abstract

Background/Objectives: GATA transcription factors play pivotal roles in regulating plant growth and development, physiological metabolism, and responses to environmental stress. However, research on *GATA* genes in sweetpotato remains limited. Methods: In this study, we identified 25 *IbGATA* genes in sweetpotato (*Ipomoea batatas* [Lam.] L.) through a genome-wide analysis. These genes were analyzed for their physicochemical properties, chromosomal localization, synteny, phylogenetic relationships, gene structure, promoter *cis-elements*, protein interaction networks, and expression profiles across various tissues and under drought stress. To elucidate the function of drought-resistant candidate genes, an in situ one-step transformation method was employed. Results: Sweetpotato *GATA* genes have a complex evolutionary history, including replication events, different selection pressures, and functional diversification. They may be involved in multiple plant stress signaling pathways. Furthermore, functional analysis revealed that *IbGATA17* enhances drought tolerance in sweetpotato by promoting proline biosynthesis and reinforcing ROS scavenging capacity. Our findings provide novel insights into the roles of *IbGATAs*, particularly *IbGATA17*, in mediating drought-stress responses in sweetpotato. Conclusions: This study provides foundational insights into the *GATA* gene family in sweetpotato and reveals the pivotal role of *IbGATA17* in simulated drought-stress response, providing a potential candidate gene for the development of drought-resistant varieties.

## 1. Introduction

Drought is considered one of the most detrimental abiotic stresses, with severe ramifications for plant growth and development [1,2]. It also imposes serious constraints on global agricultural production [3,4]. Under drought conditions, plants experience water deficit, oxidative stress, and metabolic disruption, which can result in reduced yields and compromised quality [5,6]. Sweetpotato (*Ipomoea batata* (L.) Lam.) is a globally vital crop and a crucial food source. it is rich in carbohydrates, carotenoids, vitamins, and minerals [7,8]. It plays a pivotal role in ensuring food security and combating hunger [9,10,11,12]. The crop’s remarkable adaptability and resilience to diverse planting environments, including marginal lands with poor soil conditions, contribute to its importance as a climate-resilient crop [13]. However, drought stress significantly limits sweetpotato storage root formation and storage root development, posing a serious threat to sustainable agriculture in arid and semi-arid regions [14]. Sweetpotato is a hexaploid crop with highly complex genome, complex genetic patterns, and high degree of inbreeding both within and between species [15]. Genetic engineering represents a significant method of enhancing the drought tolerance of sweetpotato. At present, some drought-tolerance-related genes have been cloned from sweetpotato, and their overexpression can enhance drought tolerance in transgenic sweetpotato plants [16,17,18,19,20,21,22].

The GATA transcription factors (TFs) are a class of type IV zinc finger DNA-binding proteins that regulate the transcription of downstream genes by binding to the W-GATA-R motif in promoter regions [23]. *GATA* family genes have been identified in multiple plant species, such as *Arabidopsis thaliana* [24], *Triticum aestivum* [25], *Capsicum annuum* and *Solanum tuberosum* [26,27], as well as *Setaria italica* [28]. In plants, GATA TFs are known to modulate diverse physiological processes, including light signaling, hormonal responses, and tolerance to abiotic stress [23]. In *A. thaliana*, GATA TFs (GNC, GNL, and B-GATA) have been shown to regulate physiological processes, including chlorophyll synthesis, flowering time, and cold resistance, and they also contribute to the balance of phototropic and gravitropic growth responses [29,30]. In rice, the GATA TFs (GATA6, GATA8, and GATA16) have been shown to exert a pivotal regulatory influence on grain size, the number of tillers, and the response to cold stress and reactive oxygen species (ROS) [31,32,33,34,35]. At present, there are few reports on *GATA* genes in sweetpotato. Overexpression of *IbGATA24* enhances the hormone signaling pathway and ROS scavenging, thereby improving the drought resistance of *A. thaliana* [22]. Wang et al. identified the *GATA* gene family in sweetpotato, however, no functional analysis of these genes was performed [36]. The molecular mechanisms through which *GATA* family genes confer drought tolerance in plants are not yet fully elucidated.

In this study, we conducted a comprehensive genome-wide analysis of the *GATA* gene family in sweetpotato, leading to the identification of *25 IbGATA* genes. Their phylogenetic relationships, gene structures, promoter cis-acting elements, and expression patterns in response to drought stress were systematically characterized. Notably, *IbGATA17* exhibited significant upregulation under drought stress. Functional analysis revealed that its overexpression enhances drought tolerance by facilitating proline biosynthesis and scavenging ROS. This provides novel insights into the molecular basis of drought tolerance in sweetpotato.

## 2. Materials and Methods

### 2.1. Plant Materials

The sweetpotato drought-resistant line ‘Xu55-2′ was utilized in the analysis of gene expression and cloning of *IbGATA17*, and the cultivar ‘Shangshu 19′ was employed for the genetic transformation and the characterization of gene functions. The sweetpotato plants were grown in a substrate made of peat and vermiculite in a 1:1 ratio. The greenhouse cultivation took place at 25 ± 3 °C, which is the natural light environment of the China Agricultural University in Beijing, China.

### 2.2. Identification of IbGATAs

We obtained the whole-genome sequences of sweetpotato from the *Ipomoea* Genome Hub (https://sweetpotao.com/: accessed on 4 May 2025) and the protein sequences of *A. thaliana* GATA transcription factors from TAIR (https://www.arabidopsis.org/: accessed on 4 May 2025). Utilizing these *A. thaliana GATAs*, a search was conducted for potential *GATA* genes in the sweetpotato genome, employing a BlastP search with an E-value cutoff of no more than 1 × 10^−5^.

To further refine our search, we retrieved the Hidden Markov Model (HMM) profile for the zinc finger domain (PF00320) from the Pfam database (now hosted by InterPro) and conducted an HMM search using TBtools-II v2.326. We merged the results from both BlastP and HMM searches and validated all candidate *GATA* genes using the CD-Search tool (https://www.ncbi.nlm.nih.gov/Structure/cdd/wrpsb.cgi: accessed on 4 May 2025) to confirm the presence of conserved domains. Finally, the remaining *GATA* genes were systematically numbered based on their chromosomal positions.

### 2.3. Protein Property Prediction and Chromosome Distribution of IbGATAs

In order to assess the physical and chemical characteristics of IbGATA proteins, such as their molecular mass, isoelectric point, instability index, and water solubility, the ExPASy ProtParam tool (https://web.expasy.org/protparam/: accessed on 4 May 2025) was employed. In addition, Cell-PLoc 2.0 (http://www.csbio.sjtu.edu.cn/bioinf/plant-multi/: accessed on 4 May 2025) was employed to predict their subcellular locations. In order to ascertain the chromosomal locations of *IbGATA* genes, genomic data from the Sweetpotato Genomics Resource (http://sweetpotato.uga.edu/index.shtml: accessed on 4 May 2025) was utilized, and the results were visualized using TBtools-II v2.326 to create an in-depth chromosomal distribution map.

### 2.4. Phylogenetic Analysis of GATAs

The amino acid sequences of the *GATAs* transcription factors from *A. thaliana* (At) and sweetpotato (*Ipomoea batatas*, Ib) were aligned using the ClustalW tool in MEGA 11.0 software [37]. The same software was used to build a phylogenetic tree following the alignment, employing the Neighbor-Joining method with a bootstrap test of 1000 [38]. The phylogenetic trees were visualized using the Interactive Tree of Life (ITOL) platform (https://itol.embl.de/index.shtml: accessed on 15 May 2025).

### 2.5. Collinearity Analysis of IbGATAs

The collinear gene pairs were predicted by a one-step MCScanX of TBtools-II v2.326 software and the resulting data were visualized using TBtools-II v2.326 [39].

### 2.6. Conserved Structural Domains and Gene Structure Analysis of IbGATAs

Ten distinct motifs within the protein sequences were pinpointed through the tried-and-true discovery process with the Multiple Em for Motif Elicitation tool, also known as MEME (https://web.mit.edu/meme/current/share/doc/overview.html: accessed on 15 May 2025). To dissect the exon–intron framework, we utilized the TBtools-II v2.326 software package in tandem with the GSDS2.0 website (https://gsds.gao-lab.org/: accessed on 15 May 2025). Our examination was grounded in the meticulous GFF annotation files.

### 2.7. Identification of Cis-Acting Elements in the IbGATAs Promoter

TBtools was used to extract 2000 bp upstream of the CDS of the *IbGATAs* gene, and then the PlantCARE tool (http://bioinformatics.psb.ugent.be/webtools/plantcare/html/: accessed on 17 May 2025) was used to analyze the cis-acting elements in the promoter region. TBtools-II v2.326 software and Python v3.7 were used to analyze the homeostatic elements in the promoter region [40]. Visualization was performed using TBtools-II v2.326 software and the Python v3.7 package Seaborn.

### 2.8. Prediction of IbGATAs Secondary Dimensional Structures

The secondary structure of the IbGATAs protein was predicted using NetSurfP-3.0 [41].

### 2.9. Protein Interaction Network of IbGATAs

The protein interaction network of the IbGATAs was analyzed on the STRING website (https://cn.string-db.org/: accessed on 23 May 2025) based on the AtGATAs proteins of *A. thaliana* [42].

### 2.10. Transcriptome and Expression Analysis

The RNA-seq data for *IbGATAs* in different sweetpotato tissues were obtained from the NCBI SRA repository (PRJCA000640) [43]. Additionally, the RNA-seq data for Xu 55-2 under PEG6000 treatment were obtained from related research [44]. Sweetpotato stem explants grown in vitro were cultured on Murashige and Skoog (MS) solid medium under 13 h of cold white light and 11 h of darkness. The 4-week-old plants were then treated with 20% polyethylene glycol (PEG) 6000 for 24 h. The sweetpotato *IbACTIN* (Genbank AY905538) gene served as an internal control and quantified gene expression by the comparative CT method. The expression of *GATA* genes (*IbGATA4*, *IbGATA8*, *IbGATA12*, *IbGATA16*, and *IbGATA17*) in sweetpotato was confirmed by quantitative reverse-transcription qRT-PCR on a 7500 Real-Time PCR instrument (Applied Biosystems, Foster City, CA, USA). The Premier 5 used for qRT-PCR primer design is listed in Supplementary Table S1. PCR cycle conditions were 95 °C for 5 min as the first denaturing step, followed by 40 cycles at 95 °C for 10 s, 60 °C for 30 min, and a gradual increase in temperature from 60 to 95 °C during the dissociation stage to monitor the specificity of each primer pair.

### 2.11. Transcriptional-Activation Assay

The coding sequence (CDS) of *IbGATA17*, as well as fragments encoding amino acids 1–74 and 75–324, were inserted into the pGBKT7 vector. The positive control was pGBKT7-53 while the negative control was pGBKT7-Lam. These constructs were transformed into the yeast strain Y2H Gold. The transformed yeast was then streaked onto SD/-Trp and SD/-Trp/-His/X-α-Gal plates and incubated at 30 °C for 2–3 days to observe growth [45].

### 2.12. Subcellular Localization of IbGATA17

The CDS of *IbGATA17*, excluding the stop codon, were inserted into pCAMBIA1300-35S-*GFP*. The construct and the control were both transfected into rice protoplasts using a polyethylene glycol–calcium-mediated method. This was followed by an 18 h incubation period to allow for transient expression. The isolation of the protoplasts and the transfection of the vectors were performed according to the method described by Yoo et al. [46]. The green and red fluorescent protein signals (GFP and RFP, respectively) were then collected using confocal laser scanning microscopy (LSM880; Zeiss, Oberkochen, Germany).

### 2.13. Production of Transgenic Plants

The CDS of *IbGATA17* was separately inserted into the pCAMBIA1300-*GFP* vector. A pair of forward and reverse highly specific fragments of the gene *IbGATA17* were inserted into the plant RNAi vector pFGC5941. The pCAMBIA1300-*IbGATA17*-*GFP* and pFGC5941-*IbGATA17* constructs were then introduced into *Agrobacterium* strain K599. The production of the *IbGATA17-OE* and *IbGATA17-Ri* plants was achieved through the utilization of Shangshu 19 cuttings employing one-step *Agrobacterium-rhizogenes*-mediated transformation. After PCR analysis of the transgenic sweetpotato storage roots, we obtained 5 overexpression and 5 RNAi lines for further study [47].

### 2.14. Drought Tolerance Assays

The WT, *IbGATA17-OE*, and *IbGATA17-Ri* plants were cultivated in flowerpots with a substrate consisting of a 1:1 mixture of peat soil and vermiculite. The PEG6000 stress treatment was performed according to the method of Zhang et al. [17], with minor modifications. Select plants with consistent growth were irrigated with Hoagland solution containing 20% PEG6000 every 4 h for a period of 48 h.

### 2.15. Measurement of Abiotic Stress Tolerance Indices

Proline, malondialdehyde (MDA), and H_2_O_2_ contents were determined using assay kits (Cominbio, Suzhou, China) following the manufacturer’s instructions using leaves of transgenic sweetpotato.

### 2.16. Statistical Analysis

All data were analyzed using a one-way ANOVA, followed by a post hoc Tukey’s test, in SPSS 27.0. Data are presented as means ± standard deviation (SD). These statistical methods were employed to analyze the gene expression and resistance-related indicators of drought tolerance. Three biological replicates were performed for each experiment.

## 3. Results

### 3.1. Characteristics of GATA Genes in Sweetpotato

The HMM profile PF00320 (GATA DNA-binding domain) from Pfam database was first employed to screen the sweetpotato proteome. Next, the GATAs protein sequence in *A. thaliana* was utilized as control and the BLASTp search was conducted on protein sequence databases of sweetpotato. After removing redundant and incomplete sequences, a total of 25 members of the *GATA* gene family were identified in the entire sweetpotato genome. The genes were separately named *IbGATA1–IbGATA25* according to their position on the chromosome. The 25 identified GATA proteins exhibited considerable diversity in their physicochemical properties. The amino acid lengths of these peptides ranged from 142 to 370 residues, the molecular weights varied from 14.87 to 94.10 kDa, and the aliphatic indices ranged from 37.05 to 73.85. Theoretical isoelectric points of the 25 GATA proteins ranged from 5.38 to 10.33. The stability of all GATA proteins was predicted, with instability indices exceeding 40. The median hydrophilicity for the 25 GATA proteins fell below zero, signifying their hydrophilic nature. Subcellular localization predictions indicated that the sweetpotato’s GATA are predominantly located within the nucleus or chloroplasts. (Table 1)

### 3.2. Chromosome Mapping of GATA Family Genes in Sweetpotato

**Figure 1:**
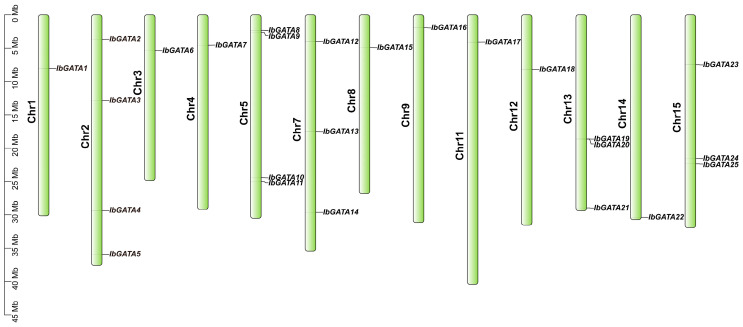
Chromosomal distribution of the *GATA* gene family in sweetpotato.

The distribution of *GATA* genes on sweetpotato chromosomes was determined based on the results of the chromosome location analysis of the 25 *GATA* family genes in sweetpotato (Figure 1). Chromosomal mapping revealed an uneven distribution of the 25 *IbGATA* genes across 13 out of the 15 sweetpotato chromosomes. Chromosomes 2 and 5 contained the largest number of *IbGATAs* (4 genes), followed by chromosomes 7, 13, and 15 (3 genes each). It is noteworthy that no *GATA* genes were identified on chromosomes 6 and 10.

The vertical bars in the figure represent chromosomes, with chromosome numbers labeled on the left. *GATA* gene names are shown on the right, and their precise positions are marked by black ticks along the chromosomes. Scale bars indicate physical distances (Mbp).

### 3.3. Cluster Analysis of GATA Family Genes in Sweetpotato

**Figure 2:**
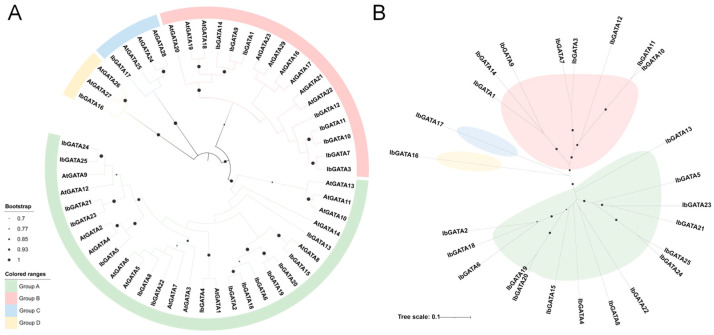
Phylogenetic analysis of IbGATA proteins. (**A**) Phylogenetic tree of IbGATAs without evolutionary rates. (**B**) Phylogenetic tree of IbGATAs with evolutionary rates. The length of branches represents the evolutionary distance.

In order to achieve complete clarification of the genetic relationship and biological function of sweetpotato *GATA* family genes, a phylogenetic tree was constructed by clustering the identified GATA family members by multiple sequence alignment (Figure 2). The classification results of the *A. thaliana* GATAs revealed the presence of four distinct subgroups, designated Groups A, B, C, and D, according to the established nomenclature. The distribution of GATAs across the phylogenetic tree was found to be uneven. The largest number of sweetpotato GATAs was found in Group A (fifteen), followed by Group B with eight. Groups C and D contained one each. A comparison of GATAs within analogous subgroups reveals a striking similarity in their structural and functional characteristics. Consequently, the biological function of the *GATA* gene in sweetpotato can be deduced from the results of analogous genes in *A. thaliana*. Phylogenetic analysis of the *GATA* gene family revealed distinct evolutionary patterns, with Group A genes (*GATA2*, *GATA18*, and *GATA6*) forming a monophyletic cluster suggestive of common ancestry through gene duplication, potentially followed by functional conservation or divergence. Notably, *GATA16* and *GATA17* exhibited elongated branches indicative of accelerated evolution, possibly due to relaxed purifying selection or positive selection for novel functions, while Group B genes (*GATA10* and *GATA11*) showed shorter branches consistent with strong functional constraints or recent duplication events. These findings collectively demonstrate the complex evolutionary history of sweetpotato *GATA* genes, involving duplication events, differential selection pressures, and functional diversification.

### 3.4. Evolutionary Dynamics of the IbGATAs Gene Family in Sweetpotato

**Figure 3:**
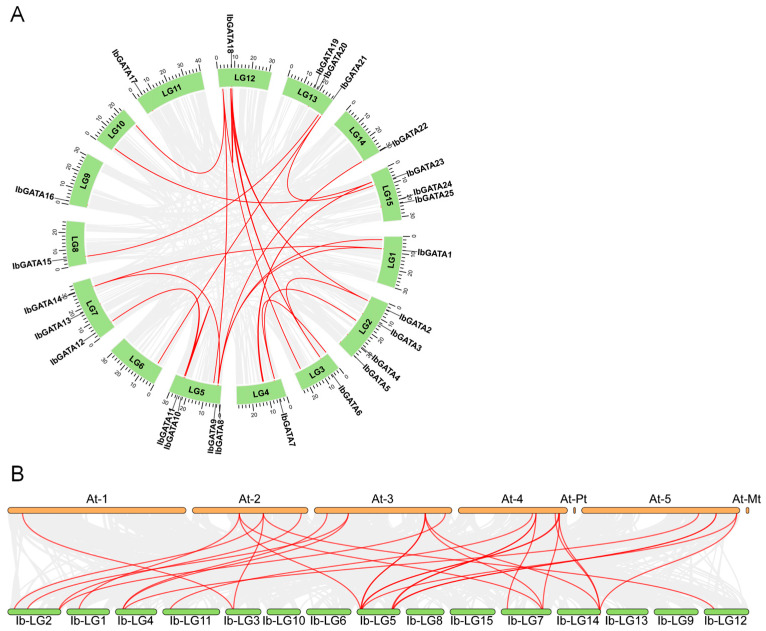
Collinearity analysis. (**A**) Syntenic relationships of *GATA* gene family in sweetpotato. The red lines represent syntenic *GATA* genes. (**B**) Interspecies collinearity of sweetpotato and *A. thaliana*. The red lines represent syntenic *GATA* genes.

To investigate the evolutionary mechanisms of the *IbGATAs* gene family in sweetpotato, a genome-wide collinearity analysis was performed. The results identified 13 collinear *IbGATAs* genes (Figure 3A). Notably, five genes (*IbGATA1*, *IbGATA9*, *IbGATA11*, *IbGATA14*, and *IbGATA18*) showed evidence of at least two duplication events, suggesting recurrent gene duplication during evolution. These duplications may have arisen through whole-genome duplication, segmental duplication, or tandem repeats, potentially driving functional diversification. The repeated duplication of these genes implies their important roles in sweetpotato evolution and development, possibly through mechanisms like neofunctionalization or subfunctionalization.

To elucidate the evolutionary mechanisms of the *IbGATA* gene family, we performed a comparative collinearity analysis between sweetpotato and *A. thaliana* (Figure 3B). Our analysis revealed 29 collinear relationships between 15 sweetpotato *IbGATA* genes (*IbGATA1*, *IbGATA2*, *IbGATA3*, *IbGATA5*, *IbGATA6*, *IbGATA7*, *IbGATA8*, *IbGATA9*, *IbGATA10*, *IbGATA11*, *IbGATA12*, *IbGATA14*, *IbGATA17*, *IbGATA18*, and *IbGATA22*) and 15 *A. thaliana GATA* genes (*AtGATA2*, *AtGATA3*, *AtGATA4*, *AtGATA5*, *AtGATA6*, *AtGATA7*, *AtGATA10*, *AtGATA13*, *AtGATA16*, *AtGATA17*, *AtGATA18*, *AtGATA19*, *AtGATA21*, *AtGATA22*, and *AtGATA25*).

### 3.5. Motifs of IbGATAs and Exon–Intron Analysis of IbGATAs Genes

**Figure 4:**
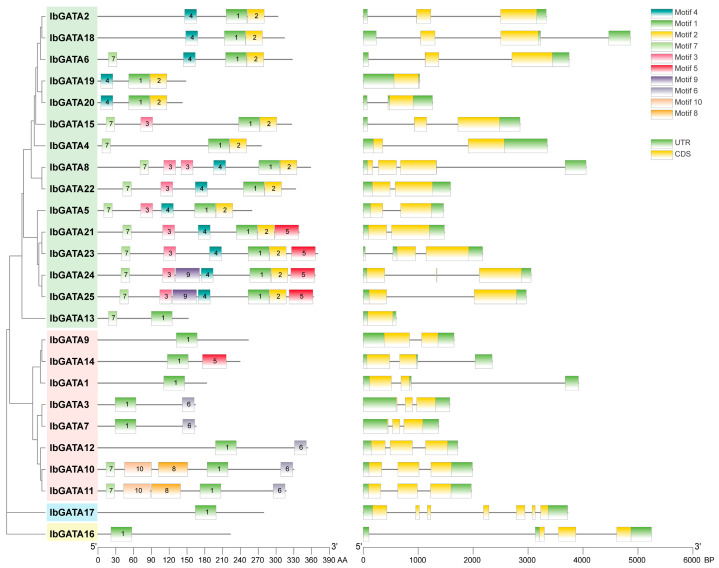
Domains and gene structure of GATAs proteins in sweetpotato.

The structural architecture of a gene, including its regulatory elements, exon–intron organization and splicing patterns, and the three-dimensional conformation of the protein it encodes, are critical determinants of protein functionality. These factors influence stability, interaction capacity, and subcellular localization [48]. The structure of genes and protein conformation are key determinants of protein function. In order to further understand the GATA protein, motif and gene structure analyses were performed. Sequence motif analysis showed that there were one to ten different motifs in the IbGATAs protein family and the distributions of motifs in different groups showed high degrees of similarity (Figure 4). All of the groups contained Motif 1, most genes in group A contained Motif 4 and Motif 7, most genes in group B contained Motif 6, and genes in groups C and D contained only motif 1. Among them, *IbGATA24* and *IbGATA25* have the most with seven motifs, while *IbGATA1*, *IbGATA9*, *IbGATA16*, and *IbGATA17* have only one motif. This suggests that Motif 1 is the most important motif for GATAs protein function and that different groups contain different conserved motifs which may be related to their different functions. These results also further illustrate the accuracy of phylogenetic analysis.

The exon–intron structures and motif compositions of sweetpotato GATA family members were subjected to visualization. An analysis of the exon–intron structures of *IbGATAs* genes was conducted, revealing a range of exon numbers from one to seven, and a range of intron numbers from one to six. Additionally, all *GATA* genes have UTR structures (Figure 4). Overall, there is significant variation in gene structures among different categories. Proteins with close phylogenetic relationships exhibit similar gene structures, such as nine out of fifteen members of group A of *GATA* genes having two exons and one intron. In group B, five out of eight genes have three exons and two introns, while the remaining two members, which are located on a separate branch of the phylogenetic tree, possess two exons and one intron. The *IbGATA17* gene contains the most exons and introns, with seven and six. It indicates that the gene structure of this gene family is relatively stable.

### 3.6. Cis-Acting Elements of IbGATA Gene Promoters

**Figure 5:**
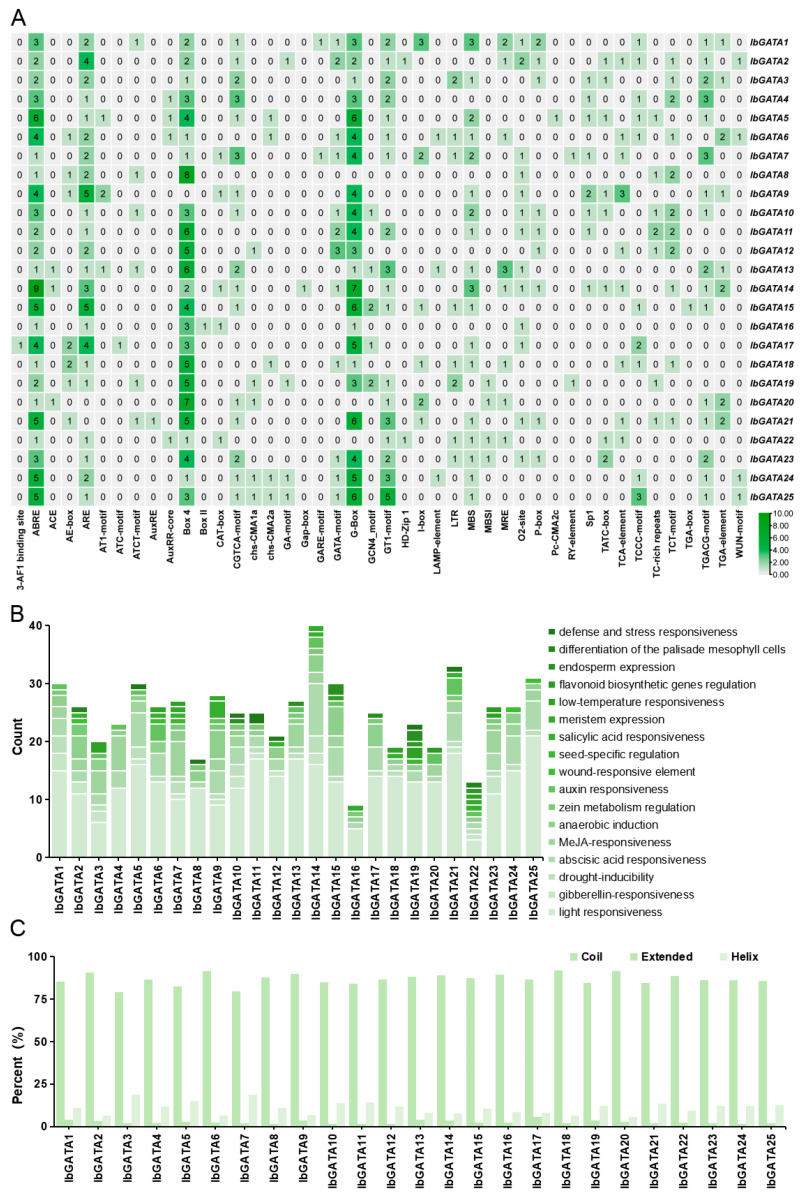
Analysis of *GATA* gene promoters. (**A**) Distribution of *cis*-acting elements in the promoters of sweetpotato *GATA* genes. The degree of green color represents the number of *cis*-acting elements upstream of the *GATAs*. (**B**) Functional categorization and number of *cis*- acting elements in the promoters of sweetpotato *GATA* genes. (**C**) Secondary structure analysis of the GATA protein.

The regulation of gene expression in hormone signals and abiotic stress responses is typically associated with factors related to the spatial distribution of relevant elements in gene promoters [2]. To elucidate the regulatory mechanisms of *IbGATA* family genes in sweetpotato growth, development, and stress responses, we employed PlantCARE for the comprehensive analysis of cis-acting elements within the 2000 base pair promoter regions of the 25 *IbGATA* genes (Figure 5A). The results showed that promoters of *GATAs* contained a variety of light-responsive elements such as G-box (TACGAT) and Box 4 (ATTAAT) and resistance-related elements include abscisic acid (ABA)-responsive element ABRE (ACGTG), antioxidant-response element ARE (AAACCA), and defense-response element WUN-motif (TAATTACTC).

Subsequently, the number of elements present in specific biological pathways was counted. The results indicated that light responsiveness was the most widely regulated feature. Following these are resistance-related elements, including abscisic acid (ABA) responsiveness, methyl jasmonate (MeJA) responsiveness, and anaerobic induction. *IbGATA14* exhibits the highest number of ABA-responsiveness elements, with a total of nine, and *IbGATA7* has the highest number of MeJA-responsiveness elements, with a total of six (Figure 5B). This suggests that *IbGATAs* may be involved in plant adversity stress pathways.

### 3.7. Significant Variations Are Evident in the Secondary Structures of GATA Proteins in Sweetpotato

In order to provide further elucidation on the structural characteristics of the GATA protein in sweetpotato, an analysis was conducted on its secondary structure. The results demonstrated that the proportion of ‘coil’ exceeded 79% for all proteins; this was the most prevalent secondary structure. The extended structures were found to be minimal, ranging from 1 to 6%. The proportion of helical structures was found to be intermediate, ranging from 6 to 19%, thus occupying a position between coil and extended structures (Figure 5C).

### 3.8. Protein Interaction Network of IbGATAs in Sweetpotato

**Figure 6:**
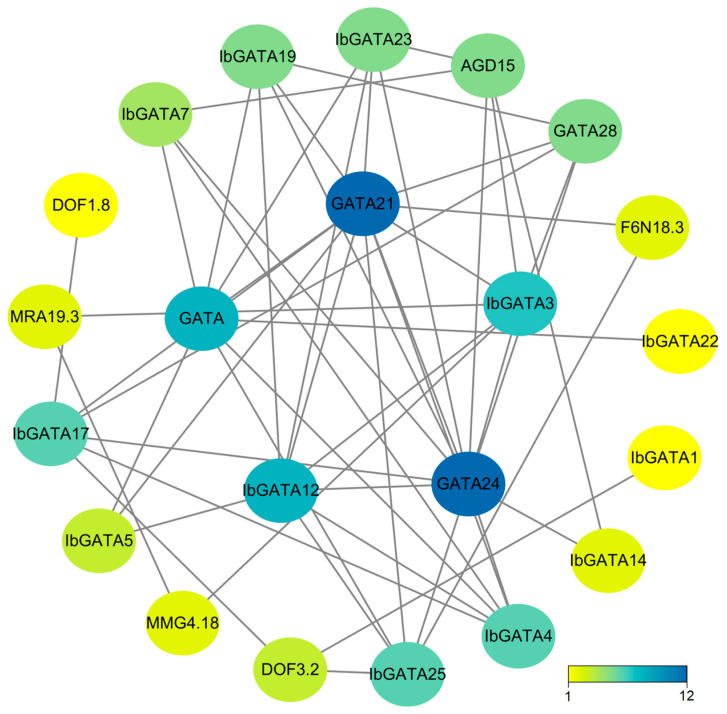
The protein interaction network of *IbGATAs*. The nodes in the figure represent proteins, with the gray lines denoting the interactions between them. The color of the nodes is indicative of the number of interacting proteins.

To delve deeper into the functions of the 25 *IbGATA* genes in sweetpotato, we utilized STRING 12.0 to compare *IbGATAs* with *A. thaliana* and identify homologous genes, thereby predicting the protein interaction network. The analysis of this network reveals that core proteins function in diverse aspects of plant biology. As indicated by protein–protein interaction (PPI) analysis, nine interactions were identified among the 25 sweetpotato GATA proteins (Figure 6). It can also interact with a variety of transcription factors. Among the proteins under consideration, *IbGATA21* and *IbGATA24* exhibit the most complex interaction networks. It has been established that there are a total of 12 interacting genes. By analyzing the protein interaction network, we can speculate that GATA proteins may play key roles in plant responses to abiotic and biotic stress. These findings provide a valuable basis for future functional studies of sweetpotato *GATA* genes.

### 3.9. Tissue-Specific Expression Analysis of IbGATAs

**Figure 7:**
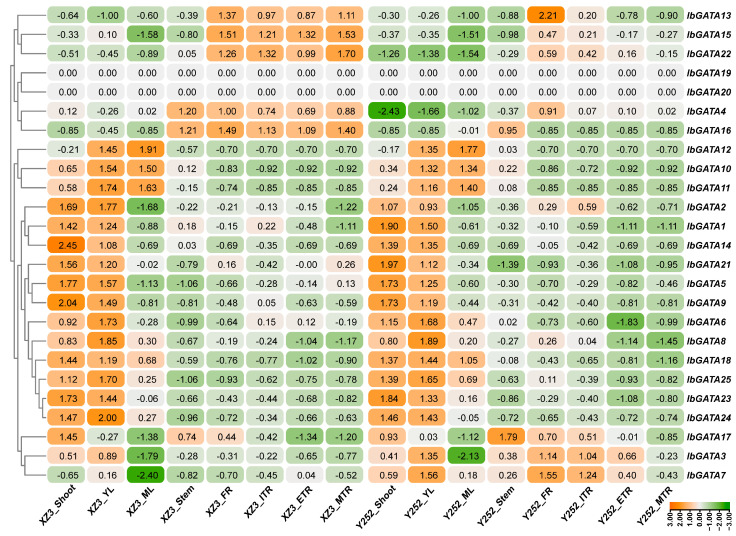
Expression levels of *IbGATAs* in different tissue from RNA-seq data. The log2 (FPKM + 1) values are displayed within the confines of the designated boxes. Higher transcript levels are shown in orange (0 to 3) and lower transcript levels are shown in green (−3 to 0).

In order to explore the biological function of *IbGATAs* in sweetpotato, the expression of *IbGATAs* was detected in eight representative sweetpotato tissues (i.e., fibrous root (FR), initial tuberous root (ITR), expanding tuberous root (ETR), mature tuberous root (MTR), stem, shoot, young leaf (YL), and mature leaf (ML)) using RNA-seq data of sweetpotato varieties Xuzi3 and Yan252. *IbGATAs* expression were observed in different sweetpotato tissues, showing tissue-specific expression in a few of the *IbGATAs* such as *IbGATA13*, *15*, and *22* (highly expressed in the FR, ITR, ETR, and MTR tissues), and *IbGATA10*, *11*, and *12* (highly expressed in YL and ML tissues) (Figure 7). Most importantly, these findings suggest that *IbGATAs* have a different pattern of expression and play a role in different tissues during the growth and development of sweetpotato.

### 3.10. The Analysis of Induction of Abiotic Stress Gene Expression

**Figure 8:**
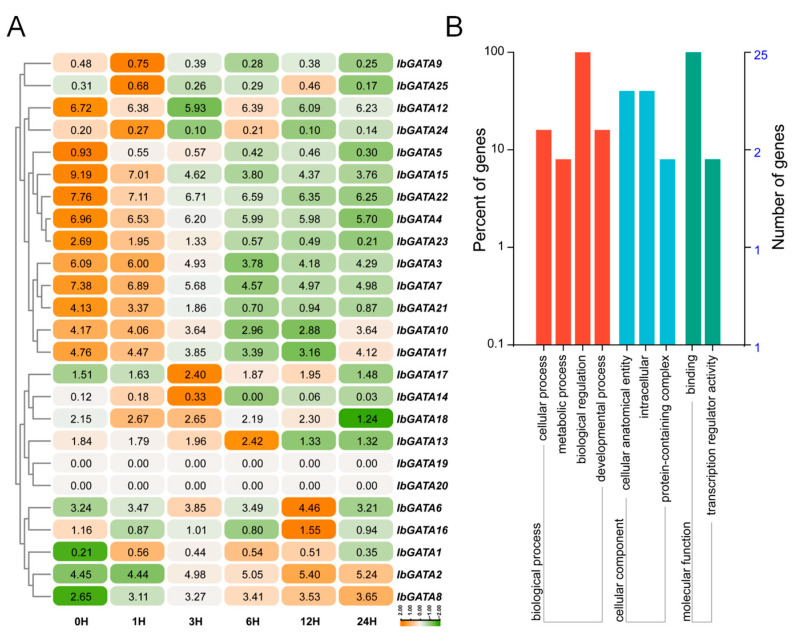
Expression analysis of *IbGATAs* under 30% PEG6000 treatment in sweetpotato. (**A**) Heat map of the expression. The log2 (FPKM + 1) values are shown in boxes. Higher transcript levels are shown in orange (0 to 2) and lower transcript levels are shown in green (−2 to 0). (**B**) GO-enrichment analysis of *IbGATA* genes differentially expressed under 30% PEG treatment. Biological process, molecular function and cellular component categories are shown.

**Figure 9:**
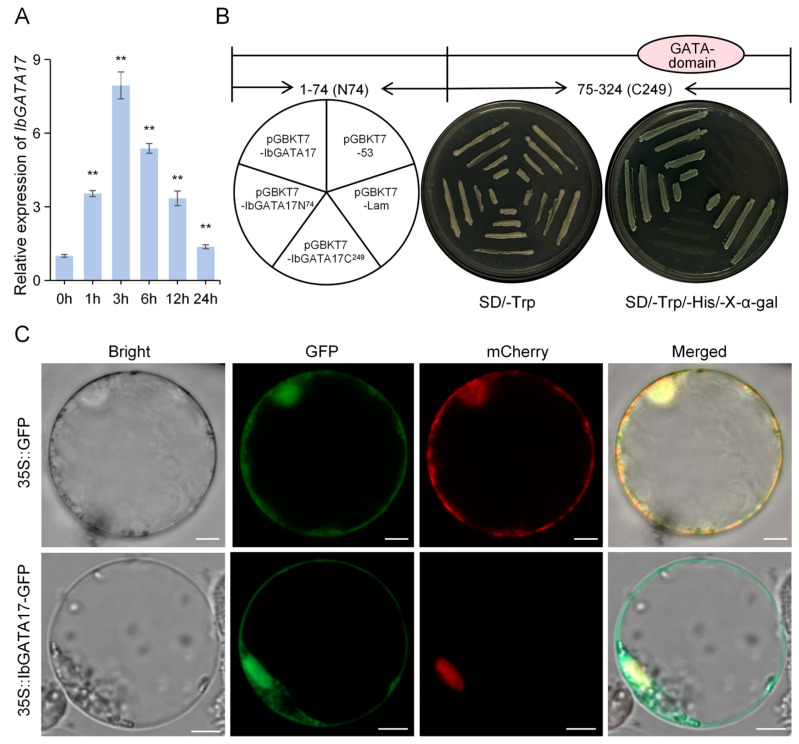
Expression analysis. Transcriptional-activation analysis and subcellular localization of IbGATA17. (**A**) Expression analysis of *IbGATA17* in sweetpotato upon PEG6000 over a 24 h period. Data are shown as mean ± SD (*n* = 3). ** indicate significant differences from that of WT at *p* < 0.01, based on Student’s *t*-test. The sweetpotato *IbACTIN* gene was used as a reference. (**B**) Transcriptional-activation assay of IbGATA17. Fusion proteins of the GAL4 DNA-binding domain and different portions of IbPIF1 were expressed in yeast strain Y2H and examined on SD/-Trp and SD/-Trp/-His/X-a-gal selection media. (**C**) Subcellular localization of IbGATA17. Rice protoplasts cells were transformed with the fusion construct (*IbGATA17*-*GFP*) and the nuclear marker *NLS*-mCherry. Bars = 5 μm.

The growth and development of plants are limited by abiotic stress. To investigate the responses of *GATA* family genes to abiotic stress, we analyzed RNA-seq data from the sweetpotato line Xu55-2 subjected to 30% PEG treatment (Figure 8A). Transcriptome analysis revealed differential expression among *IbGATA* genes, with significantly more upregulated than downregulated genes. Specifically, *IbGATA6*, *IbGATA13*, *IbGATA14*, *IbGATA16*, and *IbGATA17* were markedly upregulated under PEG treatment, whereas *IbGATA4*, *IbGATA7*, *IbGATA10*, *IbGATA15*, and *IbGATA22* were downregulated. These genes were involved in cellular progress, metabolic progress, biological progress and developmental progress, etc. (Figure 8B). Subsequently, an analysis of the gene expression was conducted by means of qRT-PCR. The results obtained were consistent with the trend observed in the transcriptome (Figure 9A and Figure S1). These results suggest that *IbGATA* genes play a role in sweetpotato’s drought tolerance, potentially regulating this adaptive process.

### 3.11. IbGATA17 Acts as a Pivotal Modulator in Drought-Stress Responses

The *IbGATA17* gene demonstrated a significant increase in expression in response to drought treatment, suggesting a potential role in enhancing plant resilience against abiotic stresses. The relative transcript levels of *IbGATA17* were quantified via qRT-PCR in sweetpotato under drought treatment. Under drought treatments, the expression levels of *IbGATA17* were upregulated nearly 7.94-fold (at 3 h) in sweetpotato (Figure 9A).

The *IbGATA17* gene demonstrated a significant increase in expression in response to drought treatment, suggesting a potential role in enhancing plant resilience against abiotic stresses. The relative transcript levels of *IbGATA17* were quantified via qRT-PCR in sweetpotato under drought treatment. Under drought treatments, the expression levels of *IbGATA17* were upregulated nearly 7.94-fold (at 3 h) in sweetpotato (Figure 9A).In order to identify potential regulators of *IbGATA17*, the gene was cloned. The protein is composed of 279 amino acids (aa) and is predicted to have a molecular weight of 30.55 kD. This is encoded by an 837-base-pair open reading frame (ORF). In order to investigate the transcription-activation activity of *IbGATA17*, three fragments encoding the full-length protein, the N-terminal 74aa residue (*IbGATA17N74*), and the C-terminal 249aa residue (*IbGATA17C249*) into the GAL4 *pGBKT7* vector were constructed. Subsequent to this, the resulting fusion constructs were then transformed into a *Y2H Gold* yeast strain (Figure 9B). The experimental results demonstrated that yeast transformants carrying BD-*IbGATA17* and BD-*IbGATA17N74* exhibited robust growth on SD/-*Trp*/-*His*/X-α-Gal medium, accompanied by the manifestation of blue fluorescence. In vivo plate assays confirmed that *IbGATA17* possesses transcription-activation activity, with its transcription-activation activity located in the N-terminal region of 74 aa residues. The present findings demonstrate that *IbGATA17* functions as a transcription activator (Figure 9B). In order to ascertain the subcellular localization of *IbGATA17*, the open reading frame of *IbGATA17* was fused with *GFP* under the control of the *35S* promoter to construct the *IbGATA17-GFP* fusion protein. The transient expression of this fusion protein in rice protoplasts was achieved via polyethylene glycol–calcium-mediated transformation. Confocal microscopy revealed that green fluorescence emitted by *IbGATA17-GFP* was localized to the nucleus and cell membrane (Figure 9C).

### 3.12. IbGATA17 Enhances Drought Tolerance of Sweetpotato

**Figure 10:**
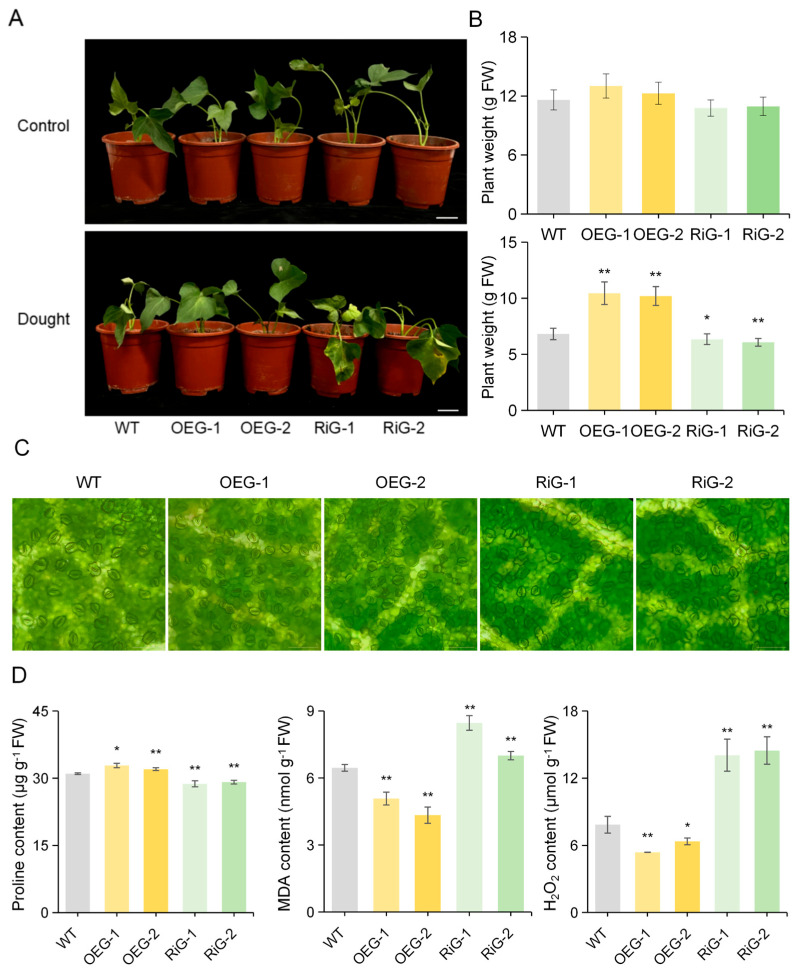
Overexpression of *IbGATA17* enhanced drought tolerance in sweetpotato. (**A**) Responses of *IbGATA17* transgenic and WT sweetpotato plants. Scale bars = 5 cm. (**B**) Plant weight grown in a flowerpot under normal conditions (control) or subjected to PEG stress for 48 h. Data are shown as mean ± SD (*n* = 3). * and ** indicate significant differences from that of WT at *p* < 0.05 and *p* < 0.01, based on Student’s *t*-test. (**C**) Stomatal aperture of the leaves of transgenic and WT plants. Scale bars = 100 μm. (**D**) Proline content, MDA content and H_2_O_2_ content in leaves of *IbGATA17* transgenic and WT plants. Data are shown as mean ± SD (*n* = 3). * and ** indicate significant differences compared to the WT at *p* < 0.05 and *p* < 0.01, based on Student’s *t*-test.

The objective of this study was to ascertain whether *IbGATA17* exerts an influence on the drought response of sweetpotato. To this end, we generated overexpressing (OE) lines (designated as OEG-1 and OEG-2) and resistance introgression (Ri) lines (designated as RiG-1 and RiG-2) via *Agrobacterium-rhizogenes*-mediated transformation for the purpose of conducting drought tolerance assays (Figure S2). In the control condition, no discernible morphological differences were observed between the transgenic and WT plants. After 48 h of PEG treatment, the *IbGATA17-OE* plants exhibited better growth, whereas their *IbGATA17-Ri* lines became wilt earlier than that of the WT plants (Figure 10A,B). We observed the stomatal openings of both transgenic and wild-type plants. We found that *IbGATA17-OE* promoted stomatal contraction, while *IbGATA17-Ri* exhibited the opposite effect (Figure 10C and Figure S3). A significantly higher level of proline content was observed in *IbGATA17-OE* than in WT plants, while lower levels were observed in *IbGATA17-Ri* (Figure 10D). Furthermore, lower levels of H_2_O_2_ and MDA accumulation were found in *IbGATA17-OE*, while higher levels were found in *IbGATA17-Ri.* These results reveal that the overexpression of *IbGATA17* activates the sweetpotato’s ROS scavenging system.

## 4. Discussion

GATA TFs are type IV zinc finger DNA-binding proteins which play a crucial role in diverse physiological processes of plant growth, development, and responses to abiotic stresses. Research has demonstrated that the *GATA* family genes are widely distributed throughout plants species, including 28 *GATA* genes in *Oryza sativa* [23], 30 in *Lycopersicon esculentum* [49], 49 in *Solanum tuberosum* [50], 64 in *Glycine max* [51], and 37 in *Zea mays* [52]. Nevertheless, the intricate workings and the core regulatory systems of *IbGATAs* remain largely unknown. Consequently, a comprehensive identification and functional study of the *GATA* gene family at the genome-wide level is imperative to enhance our understanding of their functions and the molecular intricacies underlying sweetpotato development.

In this study, a total of 25 *IbGATAs* were identified in the sweetpotato (*I. batatas*) genome. Chromosomal localization showed an uneven distribution of these genes, with chromosomes 2 and 5 harboring the highest numbers (Figure 1). Phylogenetic analysis classified these genes into four subgroups (A, B, C, and D), with subgroup A containing the largest number of genes (Figure 2). This classification is consistent with findings in *A. thaliana* [24] and *Zea mays* [52]. The distribution suggests functional diversification within the family, which is supported by the presence of conserved motifs, such as Motif 1, that appear to be critical for GATA protein function. Gene structure analysis revealed variability in exon–intron organization; notably, *IbGATA17* exhibited the most complex structure (seven exons and six introns). This structural diversity may reflect adaptive evolution, enabling *GATA* genes to acquire specialized roles in the sweetpotato.

Synteny analysis provides key insights into the mechanisms of whole-genome duplication, chromosomal rearrangement, and the functional divergence of gene families [53]. In this study, we found that there are thirteen collinear genes, including five (*IbGATA1*, *IbGATA9*, *IbGATA11*, *IbGATA14*, and *IbGATA18*) that have undergone duplication in the sweetpotato genome (Figure 3A). These results suggest that collinear genes have played a key role in the expansion and functional diversification of the GATA family in sweetpotato, potentially contributing to its adaptation to environmental stresses.

*Cis*-acting elements are defined as transcription factor binding sites that regulate the precise initiation and efficiency of gene transcription [54]. In tomato, SlHY5 suppresses the expression of *SlGATA17* by binding to the GATA-box in its promoter, thereby reducing the salt tolerance of tomato [55]. Similarly, *BdGATA13* influences primary root development in in *Arabidopsis* under gibberellic acid (GA) treatment [56]. In this study, we identified a multitude of cis-acting elements associated with abiotic stress, hormone signaling, and stress responses in the promoter regions of *IbGATAs*. These results suggest that *IbGATAs* are regulated by complex networks that integrate environmental and developmental cues (Figure 5).

Gene expression patterns are related to gene function to some extent. In wheat, *TaGATAs* showed significantly different expression patterns across different tissues and under various abiotic stress conditions [25]. Similarly, in potato, *StGATA* genes showed tissue-specific and stress-responsive expression patterns, with *StGATA12* overexpression enhancing tolerance to salinity and osmotic stresses [50]. In our study, *IbGATAs* were differentially expressed in different tissues (Figure 7) and *IbGATA6*, *IbGATA13*, *IbGATA14*, *IbGATA16*, and *IbGATA17* were significantly upregulated after PEG treatment (Figure 8A). These results suggest that different *IbGATA* genes may play different roles in different tissues and participate in drought responses to improve drought tolerance in sweetpotato.

The *GATA* family genes play a crucial role in various physiological processes during plant growth and development [29,30,31,32,33,34,57], and responses to abiotic stresses such as cold, hot, salt, and drought [35,50,58,59]. At present, there are few reports on the drought-resistance function of the *GATA* gene. Guo et al. cloned the gene *BdGATA13* in *Brachypodium distachyon* and overexpressed it in *Arabidopsis* to enhance drought tolerance [56]. Guo et al. reported that SlGATA17, which is localized in the nucleus, functions to regulate drought tolerance in tomato through the phenylpropanoid biosynthetic pathway [49]. In this study, we found that the *IbGATA17* was significantly upregulated under drought stress, with its expression increasing nearly 8-fold after 3 h of PEG treatment. This strong induction implicates *IbGATA17* as a key player in sweetpotato’s drought response (Figure 9A). Subcellular localization analysis showed that *IbGATA17* was localized in both the nucleus and the cell membrane (Figure 9C). Under simulated drought conditions, *IbGATA17* overexpression enhanced growth vigor in transgenic sweetpotato plants compared to WT, whereas *IbGATA17 RNAi* lines exhibited the opposite phenotype (Figure 10A-C). Further analysis revealed that *IbGATA17* likely enhances drought tolerance in transgenic sweetpotato by modulating stomatal regulation, proline biosynthesis, and reactive oxygen species (ROS) scavenging (Figure 10D). These results suggest that the regulatory mechanisms of *GATA17* genes in response to drought stress may differ across plant species.

Sweetpotato is a vital crop for food security, particularly in regions prone to drought. We used whole-genome identification, combined with transcriptome data analysis and qRT-PCR experiments to explore the potential drought tolerance genes in sweetpotato. At present, no *GATA17* drought tolerance reports have been reported in sweetpotato. Our identification of *IbGATA17* as a drought-responsive gene creates new opportunities for the genetic improvement of sweetpotato. Targeting *IbGATA17* or its regulatory network could enable the development of cultivars with enhanced drought tolerance, thereby contributing to sustainable agriculture in the face of climate change. While this study establishes a fundamental understanding of *IbGATA* genes in sweetpotatoes, further research is required to elucidate the precise mechanisms through which *IbGATA17* and other family members mediate stress responses. Functional studies, such as CRISPR-based gene editing or detailed protein interaction analyses, could deepen our understanding of their roles. Furthermore, investigating the interactions between *IbGATAs* and other stress-responsive pathways would be valuable.

In summary, we present genome-wide results for sweetpotato *GATA* genes, alongside analyses of their expression patterns under drought conditions. Additionally, we performed a functional analysis of *IbGATA17* and demonstrated that it can improve the drought tolerance of sweetpotato. This information could contribute to future research investigating the *IbGATAs* gene family, especially *IbGATA17* in relation to drought tolerance.

## 5. Conclusions

In this study, we used whole-genome identification and analysis techniques to elucidate the pivotal functions of the *GATA* gene family in the evolution of sweetpotato, the intricacies of their regulatory mechanisms, and their capacity for drought tolerance. The research identified genes that may be involved in the regulation of drought stress, with the *IbGATA17* gene demonstrating enhanced drought tolerance in transgenic sweetpotato plants under simulated drought conditions. These findings provide a basis for the identification of candidate genes that may be useful in the development of drought-resistant sweetpotato varieties, and they also serve as markers that can be utilized in genome-assisted crop improvement.

## Figures and Tables

**Table 1:** Information on the characterization of the identified GATA proteins.

Gene Sequence ID	Name	Number of Amino Acids (AAs)	Molecular Weight (KD)	Theoretical pI	Instability Index	Aliphatic Index	Grand Average of Hydropathicity	Localizations
*g1338*	*IbGATA1*	183	19.51	8.67	71.37	37.05	−0.737	Nucleus
*g4721*	*IbGATA2*	303	33.84	9.12	45.27	61.52	−0.64	Nucleus
*g6068*	*IbGATA3*	164	17.43	10.08	61.62	56.04	−0.858	Nucleus
*g8170*	*IbGATA4*	275	30.02	8.53	51.52	57.45	−0.735	Nucleus
*g9139*	*IbGATA5*	259	29.05	6.53	65.5	52.43	−0.833	Nucleus
*g10197*	*IbGATA6*	327	35.82	8.59	46.12	67.16	−0.452	Chloroplast
*g13467*	*IbGATA7*	165	17.15	10.33	65.1	53.94	−0.683	Chloroplast
*g16992*	*IbGATA8*	358	38.84	7.07	67.53	58.55	−0.568	Nucleus
*g17038*	*IbGATA9*	253	27.78	8.78	53.3	42.89	−0.757	Nucleus
*g19902*	*IbGATA10*	330	35.54	9.27	53.9	56.33	−0.717	Nucleus
*g19971*	*IbGATA11*	317	34.08	9.16	52.56	57.73	−0.651	Nucleus
*g25903*	*IbGATA12*	353	38.71	9.55	53.66	53.17	−0.779	Nucleus
*g27608*	*IbGATA13*	152	17.42	9.02	58.04	46.18	−1.032	Nucleus
*g29323*	*IbGATA14*	239	25.85	8.24	48.54	49.62	−0.459	Nucleus
*g31138*	*IbGATA15*	326	35.18	5.38	68.3	63.77	−0.476	Nucleus
*g34316*	*IbGATA16*	223	25.06	6.17	83.25	78.7	−0.679	Nucleus
*g42012*	*IbGATA17*	279	30.55	5.61	47.76	62.97	−0.435	Chloroplast
*g47985*	*IbGATA18*	314	34.32	8	57.89	73.85	−0.448	Nucleus
*g53671*	*IbGATA19*	148	17.05	9.71	58.17	44.8	−1.08	Nucleus
*g53673*	*IbGATA20*	142	16.21	9.61	54.09	44.01	−1.053	Nucleus
*g55280*	*IbGATA21*	339	36.91	6.26	49.21	59.09	−0.617	Nucleus
*g59772*	*IbGATA22*	333	36.75	6.99	57.43	63.78	−0.541	Nucleus
*g61042*	*IbGATA23*	370	40.13	6.01	56.75	65.73	−0.494	Nucleus
*g62825*	*IbGATA24*	366	39.81	7.01	57.65	56.42	−0.628	Nucleus
*g62945*	*IbGATA25*	363	39.48	6.3	54.38	57.19	−0.571	Chloroplast

## Data Availability

All data in this study are available in this article or in the Supplementary Materials.

## Supplementary Materials

The following supporting information can be downloaded at https://www.mdpi.com/article/10.3390/genes16101237/s1. Table S1: Sequences of the primers used in this study; Figure S1: Analysis of the gene expressions associated with PEG6000 over a 24 h period; Figure S2: Production of transgenic sweetpotato plants overexpressing and interfering with the *IbGATA17* gene; Figure S3: Stomatal aperture of the leaves.
