# Supplementary material for: Genome-Wide Identification of GATA Family Genes and Functional Analysis of IbGATA17 Under Drought Stress in Sweetpotato
Source: Genes (Basel). 2025 Oct 19;16(10):1237. doi: 10.3390/genes16101237 (PMC12563078; doi:10.3390/genes16101237)
Supplement: Supplementary file 1 [file genes-16-01237-s001.zip › genes-3908820-supplementary.pdf]

Supplementary Table S1 Sequences of the primers used in this study

	Primer name	Primer sequence（5′-3′）
Primers for qRT-PCR	IbGATA17-F	GGGTAAGGTGATGCCGTTGA
	IbGATA17-R	TTCACGGGAGAATCCGCAAA
	IbGATA4-F	CCTGTGGGTCAACCATGTGA
	IbGATA4-R	GGGAGTTTCCTCCGGAGTTG
	IbGATA8-F	AGGAGAAAACAGAGGTGCCG
	IbGATA8-R	ACTAACGCCCACCGTTTCTT
	IbGATA12-F	CAACCGAAGCAACAGCATCC
	IbGATA12-R	TAGCGTTTCTTGAAGGGCGT
	IbGATA16-F	TCAAGGAAGTGATCTGCGCC
	IbGATA16-R	AGGCCATTGGGCTTCATCTC
Primers for subcellular localization and overexpression vector	1300-IbGATA17-F	acgggggacgagctcggtaccATGGATTCAAATTTCGTCGATGA
	1300-IbGATA17-R	aagatcttcgtcgactctagaCTTGATCTCGGGTGGAGCAG
Primers for identifying Ri transgenic plants	pFGC5941-IbGATA17-UF	tttggagaggacacgctcgagATGGATTCAAATTTCGTCGATGA
	pFGC5941-IbGATA17-UR	ttaaatcatcgattgggcgcgccCGGAACTGAGAGCTCGGCG
	pFGC5941-IbGATA17-DF	aatttgcaggtatttggatccCGGAACTGAGAGCTCGGCG
	pFGC5941-IbGATA17-DR	ggtcttaattaactctctagaATGGATTCAAATTTCGTCGATGA
Primers for Transcriptional activation assay	pGBKT7-IbGATA17-F	atggccatggaggccgaattcATGGATTCAAATTTCGTCGATGA
	pGBKT7-IbGATA17-R	ccgctgcaggtcgacggatccTCACTTGATCTCGGGTGGAGC
	pGBKT7-IbGATA17-F1	atggccatggaggccgaattcTACGAGGATATTGTGCAGCTG
	pGBKT7-IbGATA17-R1	ccgctgcaggtcgacggatccCGGCTCGAGAGTCAAGGC

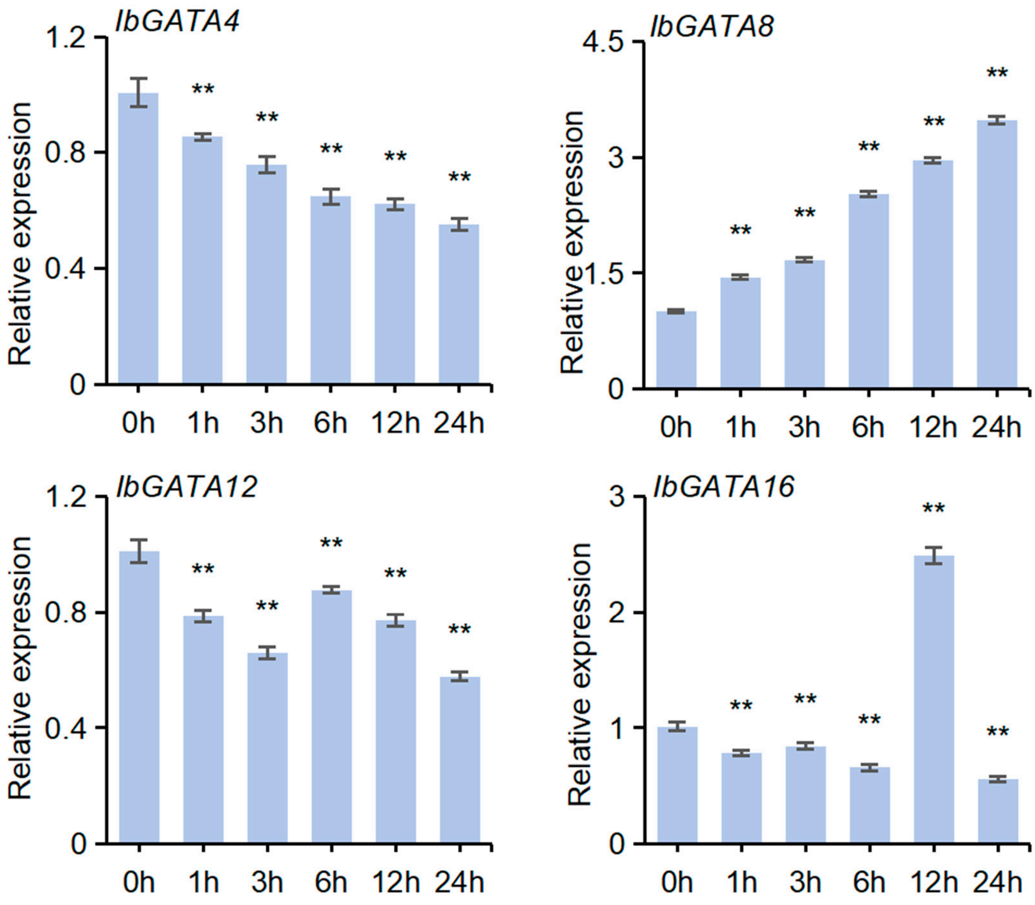

Supplemental Figure S1 Analysis the genes expression associated PEG6000 over a 24-h period. Data are shown as mean±SD (n=3). ** indicate significant differences from that of WT at P < 0.01, based on Student's t-test. The sweet potato *IbACTIN* gene was used as a reference.

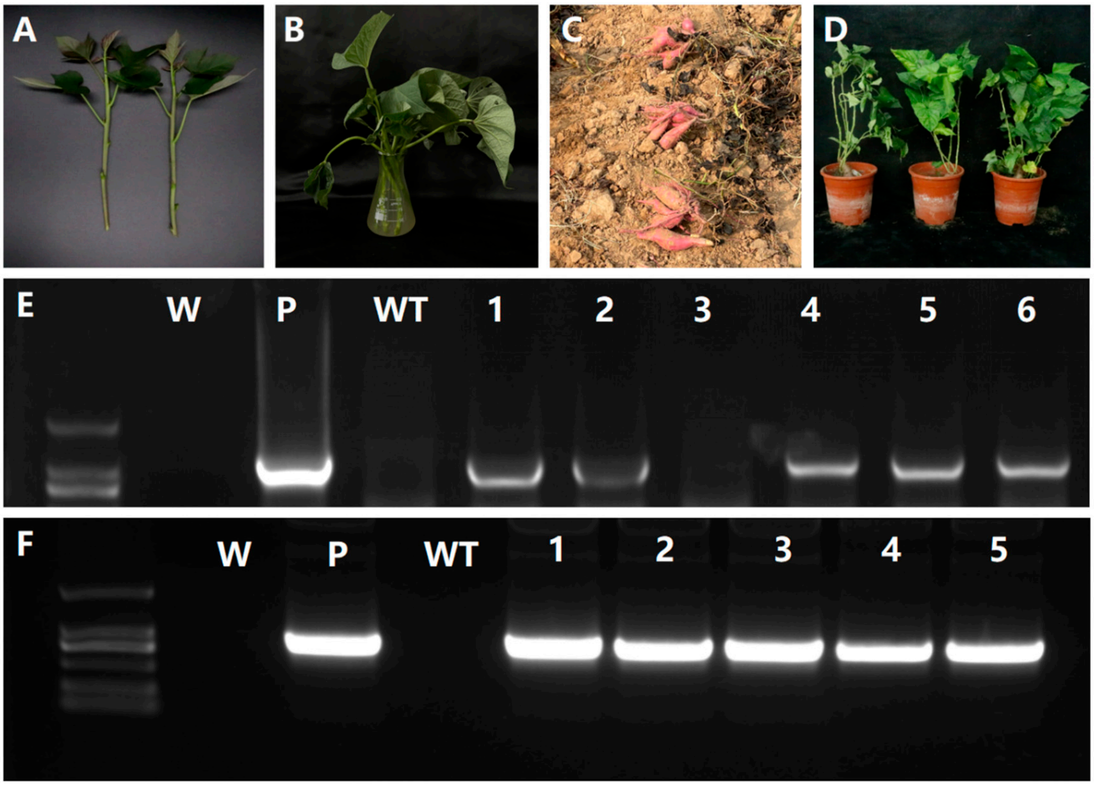

Supplemental Figure S2 Production of transgenic sweet potato plants overexpressing and interfering with the *IbGATA17* gene.

(A) Stem section of sweet potato cv. Shangshu 19.

(B) Bacterial infection.

(C) Proposed transgenic sweet potato storage roots.

(D) Plantlets of transgenic sweet potato.

(E) PCR analysis of the overexpressing transgenic plants. Lane W, water as a negative control; lane P, plasmid pCAMBIA1300 - *IbGATA17* as a positive control; lane WT, WT as a negative control; line 1,2,4,5 and 6 were positive for transgenic plants, line 3 was a negative transgenic plant.

(F) PCR analysis of the interfering transgenic plants. Lane W, water as a negative control; lane P, plasmid pCAMBIA1300 - *IbGATA17* as a positive control; lane WT, WT as a negative control; line 1,2,3,4 and 5 were positive for transgenic plants.

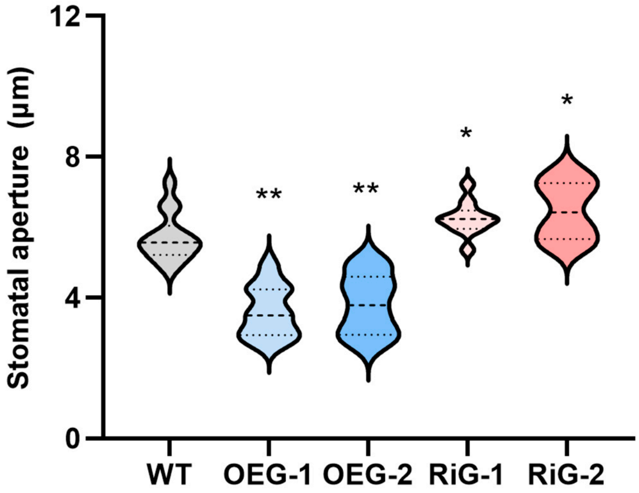

Supplemental Figure S3 Stomatal Aperture of the Leaves. * and ** indicate significant differences from that of WT at $P < 0.05$ and $P < 0.01$, based on Student's t-test.
